# The impact of COVID-19 on HIV financing in Nigeria: a call for proactive measures

**DOI:** 10.1136/bmjgh-2020-002718

**Published:** 2020-05-19

**Authors:** Tolulope Tokunyori Oladele, Babayemi Oluwaseun Olakunde, Edward Adekola Oladele, Osondu Ogbuoji, Gavin Yamey

**Affiliations:** 1 Community Prevention and Care Services Department, National Agency for the Control of AIDS (NACA), Abuja, Nigeria; 2 Infectious Diseases and Health Systems Department, FHI 360, Abuja, Nigeria; 3 Duke Center for Policy Impact in Global Health, Duke University, Durham, NC, USA

**Keywords:** health economics, health policy, health systems evaluation, public health, AIDS

**Summary box**

- COVID-19, the most devastating pandemic since the 1918 influenza pandemic, has had severe health and economic impacts, including in Nigeria.
- Nigeria has the third largest HIV epidemic worldwide.
- Despite the onset of COVID-19, efforts to control HIV cannot be abandoned.
- HIV funding must be protected from the ongoing shocks to the Nigerian economy, so that Nigeria does not lose the health gains achieved over the past decades.
- Bold proactive steps are needed, such as integrating HIV into the National Health Insurance Scheme, locking in donor commitments to HIV and building a robust health system.

## Background

The COVID-19 pandemic started in late 2019 in Wuhan province, China, and was reported on 30 December 2019 to the WHO.^1^ By 30 January 2020, WHO declared the outbreak a public health emergency of international concern.^2^ As of 4 May 2020, the WHO had documented 3 407 747 confirmed cases and 238 198 deaths worldwide.^3^

Worldwide economic disruption has resulted from COVID-19 control measures such as stay at home orders (‘lockdowns’). The United Nations Conference on Trade and Development estimates that the cost of the outbreak globally is US$2 trillion so far and predicts a global recession in 2020.^4^ The deceleration in global economic growth has demand-side causes, such as lockdowns and rising unemployment, and supply-side causes, such as a weakened global supply of many goods due to the abrupt shutdown of production lines.

Nigeria has experienced these health and economic impacts. Its first case was identified on 27 February 2020 and by 2 May 2020, there were 2170 confirmed cases and 38 deaths.^5^ The relatively low number of cases and deaths compared with countries with similar populations in Africa (e.g. Egypt) may be due to limited test kits and laboratories, under-reporting and public health control measures.

In this commentary, we describe the economic shocks that COVID-19 has caused in Nigeria, examine the HIV funding landscape and how COVID-19 may affect this landscape and propose immediate proactive steps to ensure sustainable funding for HIV control in Nigeria.

## Shocks to the Nigerian economy

Nigeria is experiencing ‘a twin shock’—a COVID-19 pandemic shock and a separate, concurrent oil price shock.^6^ Oil prices have fallen to their lowest in decades. Since Nigeria discovered crude oil, it has operated a ‘mono-economy’ with 80% of government revenue accounted for by revenue from crude oil.^7^ The price of crude oil fell to as low as $20 per barrel against the benchmark of $57 per barrel that was used to prepare the 2020 budget.^8^ Projected crude oil revenue for the year 2020 is consequently expected to fall to $13.9 billion from about $23.6 billion, and the 2020 budget deficit is expected to double from about $6 billion to $14.4 billion.^8^ The business sector and households also face dwindling income due to the restriction of movements and the closure of businesses.

These economic shocks will likely affect the financing of many different social sectors, including health. We focus on HIV/AIDS since Nigeria has the third largest epidemic in the world and a relatively high incidence rate.^9^ Nigeria’s HIV control efforts must be maintained during the COVID-19 crisis or else Nigeria could see HIV resurgence.

## Nigeria’s HIV funding landscape

**Figure 1:**
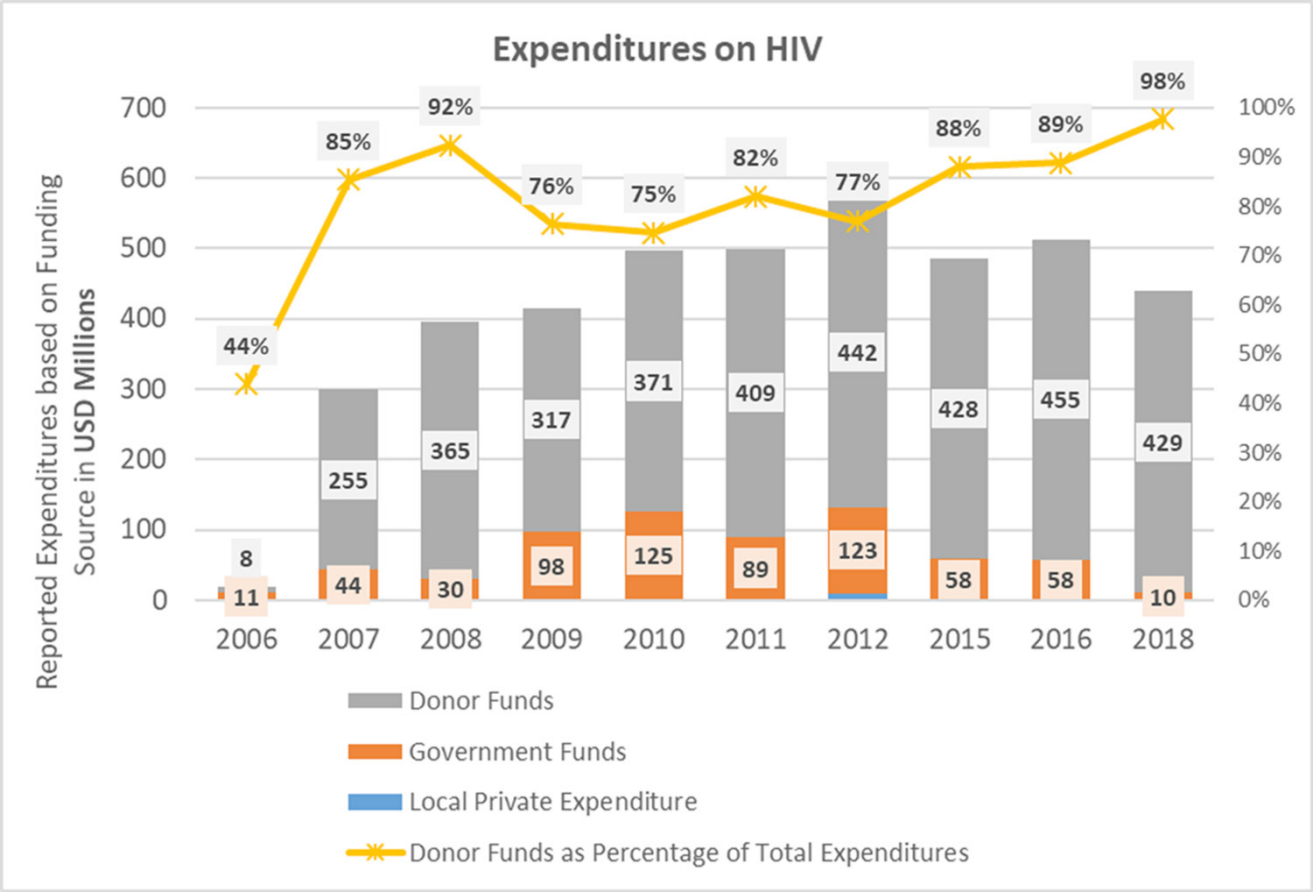
Reported HIV expenditures from all funding sources, Nigeria, 2006–2018 (adapted from UNAIDS HIV Financial Dashboard - http://hivfinancial.unaids.org/hivfinancialdashboards.html#).

Since 2006, about US$4 billion has been spent on HIV control efforts in Nigeria. About 80% of these funds have been from donors—mainly the US President’s Emergency Plan for AIDS Relief (PEPFAR) and the Global Fund to Fight AIDS, Tuberculosis and Malaria (the Global Fund) (figure 1). Firms and households contributed about 0.1%–2% of total funds, with the rest of the funds provided by the Nigerian government. Donor funds have plateaued since 2012.^10 11^ Nigeria’s recent economic growth means it will shortly lose eligibility for concessional financing.

Donor funds have largely been used for vertical programmes, rather than helping to address weaknesses in Nigeria’s health system, such as shortages of human resources, fragmentation of services and decaying infrastructure.^12 13^ The management and coordination of diseases such as HIV, tuberculosis (TB) and malaria lie outside the mainstream health system.

Nigeria’s government has been slowly working towards a more sustainable financing approach for HIV control that depends less on official development assistance (ODA).^14^ Two examples of steps taken by the government are (1) including HIV in the benefit package of social health insurance schemes, and (2) working with firms to establish a National HIV Trust Fund to increase private sector contributions to total HIV contributions.^15^

## The potential impact of COVID-19 on HIV funding

With the global and national economic effects of COVID-19, donor funding, domestic public financing and private out-of-pocket spending for HIV could all be under threat.

In the short term, donors have responded to COVID-19 by mobilising additional funding to respond to the pandemic, such as financing COVID-19 vaccine development efforts. However, in face of post-COVID-19 austerity, ODA could be one of the first spending items that get cut—for example, the 2008 global financial crisis triggered a decline in flows of ODA to low and middle-income countries.^10^

We are also seeing existing ODA flows to HIV being redirected to COVID-19. On 4 April 2020, the Global Fund released a guidance note allowing recipient countries to use HIV, TB and malaria grants to fight COVID-19 through reprogramming up to 5% of savings under existing grants and spending underused funds.^16^ In Zimbabwe, PEPFAR has instructed that $150 000 be used to buy personal protective equipment in facilities.^17^ We recognise that some of these investments (eg, contact tracing at community level) may benefit the TB/HIV response.

Donors expect that domestic public funds will be mobilised to bridge any gap in donor funding. However, with the shocks to the Nigerian economy, the government will struggle to meet its commitments to fund the budgetary allocation to HIV. If COVID-19 transmission becomes widespread throughout the country, government funds are likely to be diverted to COVID-19.

Private domestic funding for HIV will probably also fall due to declining income and loss of jobs. Half a billion people may be pushed into poverty due to COVID-19,^18^ further exacerbating the already high levels of poverty in Nigeria. Affected households may be unable to afford the cost associated with seeking HIV care in health facilities. In a shrunken economy, firms are likely to seek ways to minimise expenditure while maximising profit, which could reduce their interest in supporting the National HIV Trust Fund.

## The potential effects of reduced funding on the HIV response

A fall in funding for Nigeria’s HIV response would have short and long-term consequences.

### Short-term impacts

#### Limited access to drugs

Due to the global slowdown in drug production lines and the restriction of flights affecting logistics services, there may be a shortage of antiretroviral medicines (ARV). With the reduction in revenue and the weakened national currency (the naira), the government may not be able to raise sufficient funds to purchase ARVs. Movement restrictions in-country may result in challenges in delivering medicines and commodities to facilities.

#### Loss to follow-up

At the level of the client, limited or reduced income due to the COVID-19 shock could lead to loss to follow-up. In Nigeria, though ARVs are free to the client, clients still incur costs such as registration, laboratory and transportation, among others. Clients may default to avoid these costs.

### Long-term impacts

#### Drug resistance

With stock-outs and loss to follow-up, adherence is compromised and resistance to ARVs may develop.

#### Increased HIV incidence

Achieving viral suppression in infected people helps reduce HIV transmission.^19^ With poor adherence, the risk of transmission rises, leading to increased new infections. The gradual rise in new HIV infections noted since 2016 may therefore worsen.

#### Weakening of the health system

Dedicated HIV funding has contributed partly to strengthening the Nigerian health system, for example, funding for prevention of vertical transmission of HIV/AIDS has been used to strengthen obstetric services. A decline or withdrawal of these funds would result in a weakening of the health system.^20^

## Recommendations

Given the risks of HIV resurgence because of the COVID-19 crisis, Nigeria must take bold proactive steps to ensure sustainability of the HIV response. We make six key recommendations.

First, HIV should be quickly integrated into Nigeria’s social health insurance scheme at both state and national levels such that a pool of funds is created. This would allow for domestic resource mobilisation without further pushing people into poverty.

Second, a national COVID response plan should be developed, leveraging the existing health system where possible, filling identified gaps and providing buffer measures to accommodate the shocks to essential health services that may be imposed by COVID-19. These steps would reduce costs and ensure sustainability. Advocacy should be conducted to donors to secure their buy-in for such plans.

Third, ongoing sustainability efforts such as the HIV Trust Fund should not be abandoned. Advocacy efforts should be intensified to encourage the private sector to continue its support despite the prevailing circumstances.

Fourth, advocacy to political leaders should also be intensified to ensure that HIV remains on the national agenda. Given the risk that external assistance for HIV will fall, public financing of HIV control efforts will become more important than ever.

Fifth, Nigeria should double down in its commitment to building a robust health system with an expanded health workforce. A robust health system will help harness the benefits of economies of scale, which are required to effectively respond to ongoing and emerging epidemics.

Finally, the Nigerian government should ask donors to ‘lock in’ their commitments to HIV funding. We agree that donors should ‘protect existing official development assistance commitments, targeting support to health systems and vulnerable people in developing countries’.^21^
